# Comparison of Classifier Fusion Methods for Predicting Response to Anti HIV-1 Therapy

**DOI:** 10.1371/journal.pone.0003470

**Published:** 2008-10-21

**Authors:** André Altmann, Michal Rosen-Zvi, Mattia Prosperi, Ehud Aharoni, Hani Neuvirth, Eugen Schülter, Joachim Büch, Daniel Struck, Yardena Peres, Francesca Incardona, Anders Sönnerborg, Rolf Kaiser, Maurizio Zazzi, Thomas Lengauer

**Affiliations:** 1 Computational Biology and Applied Algorithmics, Max Planck Institute for Informatics, Saarbrücken, Germany; 2 Machine Learning Group, IBM Research Laboratory in Haifa, Haifa, Israel; 3 Department of Computer Science and Automation, University of Roma TRE, Rome, Italy; 4 Institute of Virology, University of Cologne, Cologne, Germany; 5 Retrovirology Laboratory, CRP-Santé, Luxembourg, Luxembourg; 6 Health Care and Life Sciences Group, IBM Research Laboratory in Haifa, Haifa, Israel; 7 Informa srl, Rome, Italy; 8 Division of Infectious Diseases, Department of Medicine, Karolinska Institute, Stockholm, Sweden; 9 Department of Molecular Biology, University of Siena, Siena, Italy; New York University School of Medicine, United States of America

## Abstract

**Background:**

Analysis of the viral genome for drug resistance mutations is state-of-the-art for guiding treatment selection for human immunodeficiency virus type 1 (HIV-1)-infected patients. These mutations alter the structure of viral target proteins and reduce or in the worst case completely inhibit the effect of antiretroviral compounds while maintaining the ability for effective replication. Modern anti-HIV-1 regimens comprise multiple drugs in order to prevent or at least delay the development of resistance mutations. However, commonly used HIV-1 genotype interpretation systems provide only classifications for single drugs. The EuResist initiative has collected data from about 18,500 patients to train three classifiers for predicting response to combination antiretroviral therapy, given the viral genotype and further information. In this work we compare different classifier fusion methods for combining the individual classifiers.

**Principal Findings:**

The individual classifiers yielded similar performance, and all the combination approaches considered performed equally well. The gain in performance due to combining methods did not reach statistical significance compared to the single best individual classifier on the complete training set. However, on smaller training set sizes (200 to 1,600 instances compared to 2,700) the combination significantly outperformed the individual classifiers (*p*<0.01; paired one-sided Wilcoxon test). Together with a consistent reduction of the standard deviation compared to the individual prediction engines this shows a more robust behavior of the combined system. Moreover, using the combined system we were able to identify a class of therapy courses that led to a consistent underestimation (about 0.05 AUC) of the system performance. Discovery of these therapy courses is a further hint for the robustness of the combined system.

**Conclusion:**

The combined EuResist prediction engine is freely available at http://engine.euresist.org.

## Introduction

To date 33.2 million [30.6–36.1 million] (http://www.who.int/mediacentre/news/releases/2007/pr61/en/index.html) people are estimated to be infected with the human immunodeficiency virus type 1 (HIV-1). Currently, about 20 antiretroviral compounds targeting four different stages of the viral replication cycle exist to fight the pandemic. Targets of these antiretrovirals are: entry of the virus into the host cell, reverse transcription of the viral RNA into DNA, integration of the viral genome into the host genome, and maturation of new viral particles through proteolytic processing of viral polyproteins. The group of drugs available for the longest time interrupt the process of reverse transcription. These reverse transcriptase (RT) inhibitors are subdivided in two groups. Nucleoside and nucleotide reverse transcriptase inhibitors (NRTIs) are chemically modified versions of deoxynucleosides that interfere with reverse transcription by blocking chain elongation after their incorporation into newly synthesized DNA. Instead, non-nucleoside reverse transcriptase inhibitors (NNRTIs) bind to the viral reverse transcriptase and block DNA polymerization by impairing the mobility of particular RT domains. Protease inhibitors (PIs) target the assembly of new infectious particles by occupying the active site of the viral protease that processes viral precursor polyproteins into structurally and functionally mature proteins. Compounds for preventing entry of the virus into the host cell and integration of the viral genome in the host's genome are relatively new, and not yet established or infrequently used in clinical routine.

### Antiretroviral therapy

The large variety of compounds designed for combating a single pathogen is the response to the virus' potential of escaping drug pressure by developing resistance mutations that reduce the susceptibility of the virus to the drug. Mutations leading to drug resistance are generated frequently, since the process of reverse transcription lacks a proofreading mechanism and generates single nucleotide mutations at a high rate (3.4 * 10^−5^ per nucleotide base per cycle of replication; [1]). To date, NRTIs, NNRTIs and PIs form the basis for daily routine in treating HIV patients. The current standard of care, highly active antiretroviral therapy (HAART), aims at maximally suppressing the viral replication and thus preventing or at least delaying the development of resistant variants and, thereby, the progression of the disease to AIDS and death. A typical HAART comprises three or more drugs from at least two different drug classes. Applying such a therapy can slow down the emergence of resistant variants substantially, since mutants that are resistant to all components of the regimen are unlikely to preexist, and new variants need to bring forth several escape mutations while retaining the ability of effective replication. However, treatment failure is eventually observed in most of the patients. When it occurs, clinicians face the problem of finding a new effective drug cocktail. Despite the presence of more than 20 different compounds this is a challenge, since the number of treatment options in patients failing HAART is not only reduced by resistance to the drugs administered, but also by resistance to compounds never given to the patient. This is due to the phenomenon of cross-resistance, by which a virus by becoming resistant to a drug simultaneously acquires resistance to most if not all drugs in the same drug class. Selecting an effective therapy is mandatory, because ineffective drugs lower the barrier for the virus to escape the remaining compounds and tend to result in a treatment failure after a short period.

Genotypic assays are now standard methods for guiding treatment selection by providing the genetic information of the viral strain prevalent in the patient. The information routinely extracted from the viral genome comprises all the 99 amino acid positions of the protease and the first 240 amino acid positions of the RT. Several existing tools (reviewed in [2]) support the interpretation of the complex dependence between the viral genotype and its drug susceptibility profile in vitro and in vivo. Furthermore, a reference list of resistance mutations is maintained by the International AIDS Society (IAS) [3]. Despite the availability of tools for interpreting viral resistance against single drugs there are no guidelines on how this information has to be used to rate clinical practice regimens comprising multiple compounds. Furthermore, none of the currently used genotype interpretation systems addresses the critical issue of evolution of the present virus to resistance to other drug regimens.

### The EuResist approach

The EuResist project (IST-2004-027173) aims at integrating clinical and virological data from a large number of patients and training a data-driven therapy response prediction for drug combinations rather than for single drugs. The EuResist Integrated Database (EIDB) currently comprises data from Italy (ARCA database; http://www.hivarca.net/), Germany (AREVIR database; [4]), Sweden (Karolinska Infectious Diseases and Clinical Virology Department), and Luxembourg (Retrovirology Laboratory, CRP-Santé). In order to train a classifier, response to antiretroviral therapy is dichotomized. A therapy success is defined according to clinical standards as a viral load (VL; copies of viral RNA per milliliter of blood) measure below 500 copies per milliliter or a reduction of 2 log compared to the baseline VL measure. Thus, a viral genotype and a baseline VL measure must exist at maximum three months before start of the therapy and a follow-up VL measure must be available after 8 (4–12) weeks of treatment to allow for inclusion of that therapy into the training data. Statistics of the EIDB and available training data are shown in Table 1. Three classification models for predicting response to combination antiretroviral therapy on the basis of the viral genotype and other clinical features were independently developed. The single approaches are described below. The final EuResist prediction system provides a ranking of a number of combination therapies with respect to their probability of success, given the viral genotype and additional clinical information. Basis for the ranking are the predictions of the individual classifiers. In order to provide a more reliable and robust recommendation several methods for combining the individual classifiers were investigated.

**Table 1:** Summary of the EuResist Integrated Database (release 11/2007) and training and test set.

	Patients	Sequences	VL measurements	Therapies	Successes	Failures
EIDB	18,467	22,006	240,795	64,864	-	-
Labeled Therapies	8,223	3,492	40,498	20,249	13,935	6,314
Training Set	2,389	2,722	5,444	2,722	1,822	900
Test Set	297	301	602	301	202	99

The table displays the number of Patients, Sequences, VL measurements, and Therapies for the complete EuResist Integrated Database (EIDB) and the set of therapies that could be labeled with the definition. 469 of the sequences associated with all labeled therapies belong to historic genotypes and are not directly associated with a therapy change. Moreover, detailed information on training set and test set (comprising labeled therapies with an associated sequence) is given.

### Related work

Lathrop and Pazzani [5] formulated the optimization of an anti-HIV-1 regimen with respect to drug resistance given a set of HIV-1 sequences as a triply nested combinatorial optimization problem. They presented a branch-and-bound algorithm to efficiently solve this problem. In [6] committees of Artificial Neural Networks were used to predict virological response to antiretroviral treatment. The response was not dichotomized but the actual change between baseline and follow-up VL measure was predicted. Apart from the viral genotype other clinical features were considered to enhance performance. The approach was trained and validated on a rather small set of treatment change episodes (1,150 for training, 100 for testing). In [7] the online tool geno2pheno-THEO was introduced which uses exclusively features derived from the viral genome and a quantitative notion of the probability of the virus escaping to resistance in the future, namely the genetic barrier to drug resistance, to predict success of an intended regimen. The response to antiretroviral therapy was dichotomized as well, although using a different definition leading to a set of 6,300 genotype-treatment pairs. However, none of the aforementioned approaches tried to combine multiple highly optimized classifiers for achieving a more accurate and robust prediction.

Combination of multiple classifiers has been widely discussed in literature (e.g. [8]), and is also an inherent part of novel classification methods like bagging and boosting. Previously, Sinisi et al. applied multiple regression methods for predicting HIV-1 in vitro drug resistance to one protease inhibitor [9]. Typically, different combination approaches are compared on multiple datasets to identify the most suitable method for many applications. However, the presented comparison aims specifically at finding the best combination method for prediction response to anti HIV-1 therapy. Furthermore, we present a detailed analysis in terms of performance and robustness of the complete EuResist prediction system previously introduced in [10]. Similarities to that publication are a mere necessity for ensuring autonomy of this work.

## Methods

### Individual classifiers

Three classifiers for inferring virological response to anti HIV-1 therapy were developed. One constraint on the classifiers was that they receive the viral genotype and the intended treatment as the only information, since the viral genotype is the information to which all users interested in using a decision support system must have access. However, in many cases additional information is available to the user, such as the VL, the CD4+ cell count, information on previous treatment lines and previously obtained viral genotypes, the patient's age, or the patient's risk group. In the remainder of this paper we will refer to features derived from the minimal and full set of information as minimal feature set and maximal feature set, respectively. Features that can be derived from the minimal (maximal) feature set during prediction were allowed for usage in the minimal (maximal) feature set as well. For each of the three systems a different feature selection method was applied. Although various statistical learning approaches were explored for the systems, logistic regression proved to be the most accurate method in all cases. Details on the individual engines are given in the following subsections.

#### Generative Discriminative engine

The Generative Discriminative (GD) engine applies generative models to derive additional features for the classification using logistic regression. Only a small percentage of therapies in the database (Table 1) have an associated genotype and are therefore suitable for training a classifier that is supposed to receive sequence information. However, a much larger fraction of the therapies can be labeled as success or failure on the basis of the baseline and follow-up VL measures alone, since for the labeling no viral genotype is required. The GD engine thus trains a Bayesian network on about 20,000 therapies (with and without associated genotype). The network is organized in three layers and uses an indicator for the outcome of the therapy, indicators for single drugs, and indicators for drug classes. This generative model is used to compute a probability of therapy success on the basis of the drug combination alone. This probability is used as an additional feature for the classification by logistic regression, the discriminative step of the approach. Furthermore, indicators for single drugs and single mutations are input for the logistic regression.

Indicators representing a drug class are replaced with a count of the number of previously used drugs from that class when working with the maximal feature set. In this way information about past treatments is incorporated. In addition to features from the minimal set, the maximal feature set comprises indicators for mutations in previously observed genotypes, the number of past treatment lines, and the VL measure at baseline. Correlation between single mutations and the outcome of the therapy was used to select relevant mutations for the model. A detailed description on the network's setup and the selected mutations can be found in [10].

#### Mixed Effects engine

The Mixed Effects (ME) engine explores the benefit of including second- and third-order variable interactions. Since modern regimens combine multiple drugs, binary indicators representing usage of two or three specific drugs in the same regimen are introduced. Further indicators represent the occurrence of two specific mutations in the viral genome for modeling interaction effects between them. Moreover, interactions between specific single drugs and single mutations or pairs of drugs and single mutations are represented by additional covariates. In addition to the terms modeling the mixed effects other clinical measures, demographic information, and covariates based on previous treatments are used (further information in [10]).

The large number of features (due to the mixed effects) requires a strong effort in feature selection. Thus, multiple feature selection methods were used for generating candidate feature sets. Filters and embedded methods, i.e. methods that are intrinsically tied to a statistical learning method, were applied sequentially: (i) univariable filters, such as χ^2^ with rank-sum test and correlation-based feature selection [11] , were applied to reduce the set of candidate features; (ii) embedded multivariable methods, such as ridge shrinkage [12] and Akaike information criterion (AIC) selection [13] were used to eliminate correlated features and to assess the significance of features with respect to the outcome in multivariate analysis. In multiple 10-fold cross validation runs on the training data the performance of the resulting feature sets were compared with a t-statistic (adjusted for sample overlap and multiple testing). The approach leading to the best feature set was applied on all training samples to generate the final model. Unlike the GD engine, the ME engine is based on one set of features only. Missing variables are replaced by the mean (or mode) of that variable in the training data.

#### Evolutionary engine

As stated before, one major obstacle in HIV-1 treatment is the development of resistance mutations. The Evolutionary (EV) engine uses derived evolutionary features to model the virus's expected escape path from drug therapy. The representation of viral evolution is based on mutagenetic trees. Briefly, a mutagenetic tree is reconstructed from all pairwise probabilities of defined events. Here, these events are occurrences of drug resistance mutations in the viral genome. Hence, a mixture of reconstructed mutagenetic trees represents possible evolutionary pathways towards drug resistance along with probabilities for the development of the involved mutations [14]. Using geno2pheno[resistance] [15], mutation patterns leading to complete drug resistance against a single compound can be identified. Together with the current drug resistance pattern of the virus and the probabilities of the mutagenetic trees, the likelihood of the virus remaining susceptible to that drug can be computed. This likelihood is termed the genetic barrier to drug resistance [16]. The genetic barrier to drug resistance is provided together with other features, like indicators for single drugs in the treatment and indicators for IAS mutations in the genotype. In the maximal feature set, indicators for previous use of a drug and the baseline VL measure extend this list. Interactions up to second order between indicator variables are considered as well.

The feature selection approach is based on Support Vector Machines (SVMs) with a linear kernel. The approach works in three steps: (i) optimization of the cost parameter in a 10-fold cross validation setting to maximize the area under the ROC curve (AUC); (ii) generation of 25 different SVMs by 5 repetitions of 5-fold cross validation using the optimized cost parameter; (iii) computation of the z-score for every feature. All features with a mean z-score larger than 2 were selected for the final model.

### Methods for classifier combination

In principle there are two approaches to combining classifiers, namely classifier fusion and classifier selection. In classifier fusion complete information on the feature space is provided to every individual system and all outputs from the systems have to be combined, whereas in classifier selection every system is an expert in a specific domain of the feature space and the local expert alone decides for the output of the ensemble. However, the individual classifiers described above were designed to be global experts, thus only classifier fusion methods were explored.

Methods for classifier fusion can operate on class labels or continuous values (e.g. support, posterior probability) provided by every classifier. The methods range from simple non-trainable combiners like the majority vote, to very sophisticated methods that require an additional training step. In order to find the best combination method we compared several approaches ranging from simple methods to more sophisticated ones. All results were compared to a combination that has access to an oracle telling which classifier is correct. Intuitively, the predictive performance of this oracle represents the upper bound on the performance that can be achieved by combining the classifiers. The following subsections briefly introduce the combination methods considered.

#### Non-trainable combiners

As mentioned above, there are a number of simple methods to combine outputs from multiple classifiers. The most intuitive one is a simple *majority vote*, whereby every individual classifier computes a class label (in this case success or failure) and the label that receives the most votes is the output of the ensemble. One can also combine the posterior probability of observing a successful treatment as computed by the logistic regression. This continuous measure can be combined using further simple functions: *mean* returns the mean probability of success by the three classifiers [17]; *min* yields the minimal probability of success (a pessimistic measure); *max* results in the maximal predicted probability of success (an optimistic measure); *median* returns the median probability.

#### Meta-classifiers

The use of meta-classifiers is a more sophisticated method of classifier combination, which uses the individual classifiers' outputs as input for a second classification step. This allows for weighting the output of the individual classifiers. In this work we applied *quadratic discriminant analysis (QDA)*, *logistic regression*, *decision trees*, and *naïve Bayes* (operating on class labels) as meta-classifiers.

#### Decision templates and Dempster-Shafer

The decision template combiner was introduced by Kuncheva [18]. The main idea is to remember the most typical output of the individual classifiers for each class, termed decision template. Given the predictions for a new instance by all classifiers the class with the closest (according to some distance measure) decision template is the output of the ensemble.

Let ***x*** be an instance, then *DP* ***_x_*** is the associated decision profile. The decision profile for an instance contains the support (e.g. the posterior probability) by every classifier for every class. Thus, *DP* ***_x_*** is an *I×J*-matrix, where *I* and *J* correspond to the number of classifiers and classes, respectively. The decision template combiner is trained by computing the decision templates *DT* for every class. The *DT* for the class *ω_j_* is simply the mean of all decision profiles for instances ***x*** belonging that class. Hence,
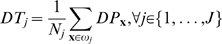
where *N_j_* is the number of elements in *ω_j_* . For a new sample, the corresponding decision profile is computed and compared to the decision templates for all classes using a suitable distance measure. The class with the closest decision template is the output of the ensemble. Thus, the decision template combiner is a nearest-mean classifier that operates on decision space rather than on feature space. We used the squared Euclidean distance to compute the support for every class:
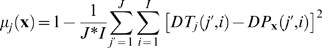
where *DT_j_ (j′, i)* is the *(j′, i)*th entry in *DT_j_* . Decision templates were reported to outperform other combiners (e.g. [18] and [19]).

Decision templates can also be used to compute a combination that is motivated by the evidence combination of the Dempster-Shafer theory. Instead of computing the similarity between a decision template and the decision profile, a more complex computation is carried out as described in detail in [20]. We refer to these two methods as *Decision Templates* and *Dempster-Shafer*, respectively.

#### Clusters in decision space

Regions in decision space where the classifiers disagree on the outcome are of particular interest in classifier combination. Therefore, we propose the following method that finds clusters in decision space and learns separate logistic regression models for every cluster for fusing the individual predictions. Let *s_i_* be the posterior probability of observing a successful treatment predicted by classifier *i*. Then we express the (dis)agreement between two classifiers by computing:
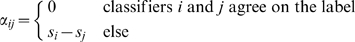
for all *i* and *j* where *i<j* . Thus, in case of disagreement between two classifiers, the computed value expresses the magnitude of disagreement. These agreements are computed for all instances of the training set and used as input to a *k*-medoid clustering. For all resulting *k* clusters an individual logistic regression is trained on all instances associated with the cluster using the *s_i_* as input. The idea is that in clusters where e.g. classifier 1 and 2 agree, and classifier 3 tends to predict lower success probabilities the logistic regression can either increase or decrease the influence of classifier 3, depending on how often predictions by that classifier are correct or incorrect, respectively.

When a new instance has to be classified then first the agreement between the classifiers is computed for locating the closest cluster. In a second step the logistic regression associated with that cluster is used to calculate the output of the ensemble. The number of clusters *k*, the only parameter of this method, is optimized in a 10-fold cross validation. The approach is motivated by the *behavior knowledge space (BKS)* method [21], which uses a look-up table to generate the output of the ensemble. However, the BKS method is known to easily over train, and does not work with continuous predictions.

#### Local accuracy-based weighting

Woods et al. [22] propose a method that uses one *k*-nearest-neighbor (knn) classifier for every individual classifier to assess the local accuracy of that classifier given the input features. The output is solely given by the most reliable classifier of the ensemble. Since the three classifiers in this setting are trained to be global experts, we applied the proposed method to compute the reliability estimate for each classifier given the features of an instance. In contrast to the method proposed by Woods et al. [22], the output is a weighted mean based on these reliability estimates.

In order to use a knn classifier as a reliability estimator the labels from the original instances are replaced by an indicator of whether the classifier in question was correct on that instance or not. With the replaced labels and the originally used features the knn classifier reports the fraction of correctly classified samples in the neighborhood of the query instance. This fraction can be used as a local reliability estimator. The output by the ensemble is then defined by a weighted mean:
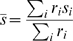
where *s_i_* and *r_i_* are the posterior probability of observing a successful treatment and the local accuracy for classifier *i*, respectively. For simplicity, only Euclidean distance was used in the knn classifier, the number of neighbors *k* was optimized in a 10-fold cross validation setting.

#### Combining classifiers on the feature level

As described above, every individual classifier uses a different feature set, specifically, different derived features, but the same statistical learning method. Thus, a further combination strategy is the use of all features selected for the individual classifier as input to a single logistic regression rather than computing a consensus of the individual classifiers' predictions.

### Data

About 3,000 instances in the EIDB met the requirements for use as a learning instance. From this complete set, 10% of the data were randomly set aside and used as an independent test set (Table 1). The split of the training data in 10 equally sized folds was fixed, allowing for 10-fold cross validation of the individual classifiers. The same 10 folds were used for a 10-fold cross validation of the combination approaches. Classification performance was measured as accuracy (i.e. the fraction of correctly classified examples) and the area under the receiver operating characteristics curve (AUC). Briefly, the AUC is a value between 0 and 1 and corresponds to the probability that a randomly selected positive example receives a higher score than a randomly selected negative example [23]. Thus, a higher AUC corresponds to a better performance.

## Results

Results for the individual classifiers using the minimal and maximal feature set are summarized in Table 2. The use of the extended feature set significantly improved the performance of the GD and EV engine with respect to the AUC (*p* = 0.001953 for both) using a paired Wilcoxon test. With respect to accuracy only the improvement observed by the EV engine reached statistical significance (*p* = 0.006836). Remarkably, replacement of all missing additional features in the case of the ME engine when working with the minimal feature set did not result in a significant loss in performance (*p* = 0.3125 and *p* = 0.3120 with respect to AUC and accuracy, respectively).

**Table 2:** Results for the individual classifiers on training set and test set.

Engine	minimal feature set				maximal feature set			
	AUC		Accuracy		AUC		Accuracy	
	Train	Test	Train	Test	Train	Test	Train	Test
GD	0.747 (0.027)	0.744	0.745 (0.024)	0.724	0.768 (0.025)	0.760	0.752 (0.028)	0.757
ME	0.758 (0.019)	0.745	0.748 (0.031)	0.757	0.762 (0.021)	0.742	0.754 (0.030)	0.757
EV	0.766 (0.030)	0.768	0.754 (0.031)	0.748	0.789 (0.023)	0.804	0.780 (0.032)	0.751

The table displays the performance, measured in AUC and Accuracy, achieved by the individual classifiers on the training set (using 10-fold cross validation; standard deviation in brackets) and the test set using different feature sets.

### Correlation among classifiers

The performances of the individual classifiers were very similar. Pearson's correlation coefficient (*r*) indicated that the predicted probability of success for the training instances using the minimal (maximal) feature set were highly correlated (i.e. close to 1): GD-ME 0.812 (0.868); GD-EV 0.797 (0.786); ME-EV 0.774 (0.768). In fact, the three classifiers agreed on the same label in 80.4% (81.7%) of the cases using the minimal (maximal) feature set. Notably, agreement of the three classifiers on the wrong label occurred more frequently in instances labeled as failure than in instances labeled as success (39% vs. 4% and 37% vs. 4% using the minimal and maximal feature set, respectively; both p<2.2 * 10^−16^ with Fisher's exact test).

This behavior led to further investigation of the instances labeled as failure in the EIDB. Indeed, 145 of 350 failing instances, which were predicted to be a success by all three engines, achieve a VL below 500 copies per ml once during the course of the therapy. However, this reduction was not achieved during the time interval that was used in the applied definition of therapy success. Among the remaining 550 failing cases this ocurred only 100 times. Using a Fisher's exact test this difference was highly significant (*p* = 4.8*10^−14^). These results were qualitatively the same when using the maximal feature set.

### Results of combination methods

**Figure 1:**
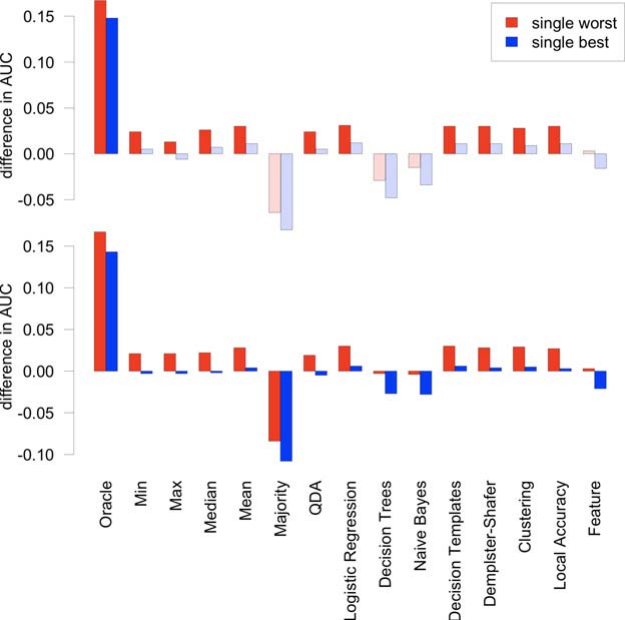
Improvement in AUC of combination methods compared to the single best and single worst classifiers. The figure displays the improvement in AUC of all combination methods over the single best (blue bars) and single worst (red bars) classifiers on the training set (upper panel) and the test set (lower panel). Significance of the improvement on the training set was computed with a one-sided paired Wilcoxon test. Solidly colored bars indicate significant (at a 0.05 *p*-value threshold) improvements, as opposed to lightly shaded bars for insignificant improvements. On the test set no *p*-values could be computed.

Table 3 summarizes the results achieved by combining the individual classifiers, and Figure 1 depicts the improvement in AUC on training and test set of the combination methods compared to the single best and single worst classifier, respectively. Most combination methods improved performance significantly over the single worst classifier. However, only the oracle could establish a significant improvement over the single best classifier. Overall, performances of the combination approaches were quite similar. The pessimistic *min* combiner yielded better performance than the optimistic *max* combiner. Among the non-trainable approaches tested, the *mean* combiner yielded the best performance. The logistic regression was the best performing meta-classifier. In fact, the logistic regression can be regarded as a weighted mean, with the weights depending on the individual classifier's accuracy, and the correlation between classifiers. Moreover, using all features of the individual classifiers as input to a single logistic regression did not improve over the single best approach.

**Table 3:** Results for the combined classifiers on training and test set.

Method	minimal feature set				maximal feature set			
	AUC		Accuracy		AUC		Accuracy	
	Train	Test	Train	Test	Train	Test	Train	Test
Single Best	0.766 (0.030)	0.768	0.754 (0.031)	0.748	0.789 (0.023)	0.804	0.780 (0.032)	0.751
Oracle	0.914 (0.015)	0.911	0.842 (0.025)	0.844	0.917 (0.013)	0.920	0.850 (0.022)	0.860
Min	0.771 (0.020)	0.765	0.746 (0.027)	0.761	0.792 (0.021)	0.793	0.760 (0.030)	0.764
Max	0.760 (0.023)	0.765	0.742 (0.030)	0.731	0.779 (0.021)	0.779	0.757 (0.030)	0.741
Median	0.773 (0.020)	0.766	0.759 (0.027)	0.766	0.789 (0.029)	0.786	0.768 (0.029)	0.761
Mean	0.777 (0.020)	0.772	0.760 (0.024)	0.744	0.794 (0.019)	0.793	0.780 (0.028)	0.781
Majority	0.683 (0.023)	0.660	0.759 (0.027)	0.738	0.697 (0.027)	0.683	0.768 (0.029)	0.761
QDA	0.771 (0.020)	0.763	0.755 (0.031)	0.738	0.790 (0.022)	0.794	0.769 (0.027)	0.764
Logistic Regression	0.778 (0.021)	0.774	0.762 (0.028)	0.744	0.798 (0.020)	0.805	0.781 (0.030)	0.771
Decision Trees	0.718 (0.044)	0.741	0.748 (0.032)	0.757	0.722 (0.033)	0.678	0.777 (0.032)	0.757
Naïve Bayes	0.732 (0.027)	0.740	0.759 (0.027)	0.738	0.752 (0.028)	0.753	0.768 (0.029)	0.761
Decision Templates	0.777 (0.021)	0.774	0.755 (0.027)	0.754	0.796 (0.019)	0.797	0.766 (0.026)	0.767
Dempster-Shafer	0.777 (0.021)	0.772	0.755 (0.024)	0.754	0.796 (0.019)	0.796	0.767 (0.026)	0.764
Clustering	0.775 (0.019)	0.773	0.758 (0.029)	0.741	0.797 (0.018)	0.800	0.783 (0.028)	0.784
Local Accuracy	0.777 (0.020)	0.771	0.761 (0.025)	0.741	0.795 (0.019)	0.791	0.781 (0.029)	0.777
Feature	0.750 (0.026)	0.747	0.745 (0.029)	0.751	0.786 (0.021)	0.779	0.780 (0.029)	0.767

The table summarizes the results achieved by the different combination approaches on the training set (10-fold cross validation; standard deviation in brackets) and the test set. The reference methods are *Single Best* and *Oracle*, the non-trainable combiners are named according to their function, the meta-classifiers according to the statistical learning methods. *Decision Templates*, *Dampster-Shafer*, *Clustering* and *Local Accuracy* are the methods described in detail in the Methods section. *Feature* is the combination on the feature level.

**Figure 2:**
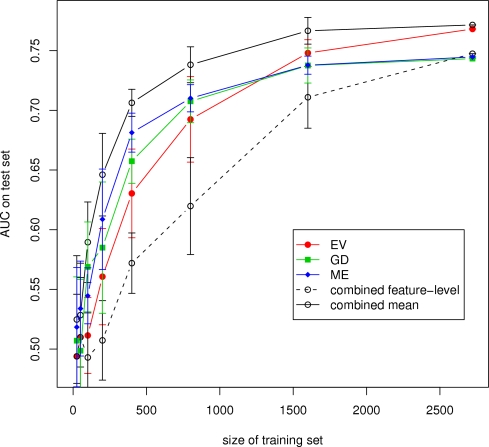
Learning curves for the individual classifiers, the mean combiner, and the combination on feature level. The figure shows the development of the mean AUC on the test set depending on the amount of available training data for the individual classifiers, the mean combiner, and the combination on the feature level using the minimal feature set. Error bars indicate the standard deviation on 10 repetitions.

Figure 2 shows the learning curves for the three individual classifiers, the mean combiner, and the combiner on the feature level. The curves depict the mean AUC (after 10 repetitions) on the test set achieved with varying sizes of the training set (25, 50, 100, 200, 400, 800, 1600, 2722). In every repetition the training samples were randomly selected from the complete set of training instances. The mean combiner appeared to learn faster and significantly outperformed the single best engine with a training set size of 200 samples (*p* = 0.009766 with a paired one-sided Wilcoxon test). The improvement remained significant up to a training set size of 1,600 samples (*p* = 0.001953). The combination on feature level was significantly (*p* = 0.001953) worse than the worst single approach for all training set sizes (except for complete set).

### Impact of *ambiguous* failures

In order to further study the impact of *ambiguous* failures (i.e. instances labeled as failure but achieving a VL below 500 cp per ml once during the course of treatment) on the performance of the individual classifiers and the combination by mean or on the feature level, they were removed from the training set, the test set, or both sets. After removal the classifiers were retrained and tested on the resulting new training and test set, respectively. The results in Table 4 suggest that training with the *ambiguous* failures does not impact the classification performance (columns “none” vs. “only train”, and columns “only test” vs. “both”). However, the *ambiguous* cases have great impact on the assessed performance. Removal of these cases increases the resulting AUC by 0.05.

**Table 4:** Results on the (un)cleaned test set when individual classifiers are trained on the (un)cleaned training set.

Engine	minimal features set				maximal feature set			
	*ambiguous* instances removed from				*ambiguous* instances removed from			
	none	only train	only test	both	none	only train	only test	both
GD	0.744	0.738	0.784	0.786	0.760	0.747	0.808	0.806
ME	0.745	0.739	0.770	0.771	0.742	0.757	0.808	0.810
EV	0.768	0.776	0.811	0.824	0.804	0.812	0.846	0.855
Mean	0.772	0.767	0.812	0.814	0.793	0.791	0.849	0.849
Feature	0.747	0.754	0.797	0.808	0.779	0.787	0.832	0.842

The table summarizes the results, measured in AUC for the individual classifiers, the mean combiner, and the combination of feature level when retrained on the (un)cleaned training set and tested on the (un)cleaned test set. Cleaned refers to the removal of *ambiguous* failing instances.

However, there might still be an influence of these *ambiguous* failures on the performance of the trainable combination methods. For verification we removed these cases whenever performance measures were computed (also in 10-fold cross validation) and trained a selection of the combination methods on the complete training data and on the cleaned training data. The results in Table 5 suggest that the trainable combination methods were not biased by the *ambiguous* failures.

**Table 5:** AUC for the combined engines on training set and test set with the *ambiguous* cases removed from test set and training set or test set only.

Method	minimal feature set		maximal feature set	
	Train	Test	Train	Test
removed from test only				
Single Best	0.809 (0.021)	0.811	0.839 (0.017)	0.847
Oracle	0.935 (0.012)	0.936	0.945 (0.014)	0.950
Min	0.817 (0.019)	0.807	0.847 (0.022)	0.848
Max	0.807 (0.024)	0.810	0.832 (0.018)	0.824
Median	0.820 (0.020)	0.810	0.844 (0.021)	0.835
Mean	0.823 (0.019)	0.816	0.850 (0.019)	0.847
Logistic Regression	0.824 (0.019)	0.816	0.852 (0.017)	0.856
Decision Templates	0.823 (0.018)	0.818	0.851 (0.019)	0.850
Clustering	0.822 (0.020)	0.808	0.852 (0.017)	0.850
Local Accuracy	0.823 (0.019)	0.813	0.850 (0.019)	0.844
removed from train and test				
Logistic Regression	0.825 (0.019)	0.816	0.852 (0.017)	0.856
Decision Templates	0.823 (0.018)	0.818	0.851 (0.019)	0.850
Clustering	0.822 (0.021)	0.796	0.852 (0.017)	0.844
Local Accuracy	0.823 (0.019)	0.813	0.850 (0.019)	0.843

The table displays the results, measured in AUC, on training set (10-fold cross validation; standard deviation in brackets) and test set for a selection of combination approaches when trained on the (un)cleaned training set. For computation of the AUC the *ambiguous* cases were always removed.

A possibility for circumventing the (artificial) dichotomization of virological response is the prediction of change in VL between the baseline value and the measurement taken at the follow-up time point. Logistic regression was replaced by linear regression in the individual classifiers for predicting the change in VL. Using the maximal feature set the GD (ME, EV) engine achieved a correlation (*r*) of 0.658±0.023 (0.664±0.023, 0.679±0.020) on the training set [10]. The mean combiner yielded a correlation of 0.691±0.019. However, the oracle achieved *r* = 0.834±0.012. Although small, the difference between EV and the mean prediction reached statistical significance (*p* = 0.004883) using a one-sided paired Wilcoxon test. Results on the test set were qualitatively the same: GD (ME, EV) reached a correlation of 0.657 (0.642, 0.678) and the mean combiner (oracle) reached 0.681 (0.814).

## Discussion

The performance of the methods considered for combining the individual classifiers improved only little over the single best method on both sets of available features. It turns out that the simple non-trainable methods perform quite well, especially the mean combiner. This phenomenon has been previously discussed in literature (e.g. [18] and [24]). Here we focused on finding the best combination strategy for a particular task. The advantage of the mean combiner is that it does not require an additional training step (and therefore no additional data), although it ranges among the best methods studied. Moreover, this combination strategy is easy to explain to end-users of the prediction system.

The learning curves in Figure 2 show that the mean combiner learns faster (gives more reliable predictions with fewer training data) than the individual prediction systems. Moreover, the curves show that the combined performance is not dominated by the single best approach as the results on the full training set might suggest. Furthermore, the learning curve for the combination on the feature level indicates that more training data is needed to achieve full performance. In general, combining the three individual approaches leads to a reduction of the standard deviation for almost all combination methods. This suggests a more robust behavior of the combined system.

In the cases of failing regimens, all three classifiers very frequently agree upon the wrong label, precisely in 350 of 900 (39%) failing regimens in the training data using the minimal feature set. There are two possible scenarios why the VL drop below 500 copies per ml did not take place during the observed time interval despite the concordant prediction of success by all the three engines:

1. Resistance against one or more antiretroviral agents is not visible in the available baseline genotype but stored in the viral population and rapidly selected, which would lead to an initial decrease in VL shortly after therapy switch, and a subsequent rapid increase before the target time frame.
2. The patient/virus is heavily pretreated and therefore it takes longer to respond to the changed regimen, or the patient is not completely adherent to the regimen, both cases lead to a delayed reduction in VL after the observed time frame.

**Figure 3:**
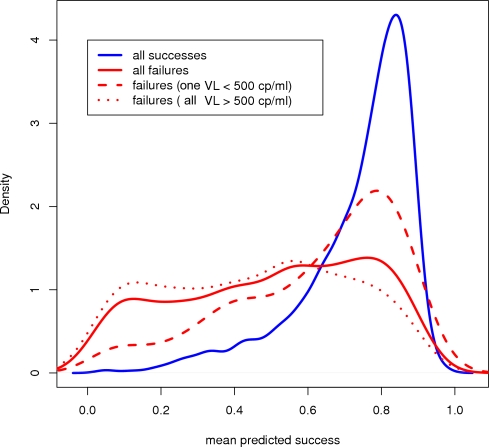
Distribution of predicted success probabilities. Distribution of the predicted success for all successful therapies (blue solid), all failing therapies (red solid), failing therapies with at least one VL measure below 500 during the regimen (red dashed), and failing therapies with all VL measures above 500 (red dotted) of the mean combiner using the minimal feature set.

Figure 3 shows the distribution of predicted success provided by the mean combiner using the minimal feature set. There is a clear peak around 0.8 for instances labeled as success whereas the predictions for the failing cases seem to be uniformly distributed. Interestingly, the distribution of the failing cases with a VL below 500 copies per ml resembles more the distribution for success than for failure.

The approach to predicting the change in VL exhibited moderate performance. In general, the task of predicting change in VL is harder, since many host factors, which are not available to the prediction engines, contribute to the effective change in individual patients. However, guidelines for treating HIV patients recommend a complete suppression of the virus below the limit of detection [25]. Thus, dichotomizing the outcome and instead solving the classification task is an adequate solution, since classifiers can be used for computing the probability of achieving complete suppression.

### Conclusion

The use of the maximal feature set consistently outperformed the minimal feature set in the combined system. Among the studied combination approaches the logistic regression performed best, although not significantly better than the mean of the individual classifications. The mean is a simple and effective combination method for this scenario. Variations in the size of the training set showed that a system combining the individual classifiers by the mean achieves better performance with fewer training samples than the individual classifiers themselves or a logistic regression using all the features of the individual classifiers. This and the consistent reduction of the standard deviation of the performance measures lead to the conclusion that the mean combiner is more robust than the individual classifiers, although the performance is not always significantly improved. Moreover, the mean is a combination strategy that is easily explainable to the end-users of the system.

In this study we discovered *ambiguous* failures. These therapies are classified as failure but have a VL measurement below 500 copies per ml. Although these instances did neither significantly influence the learning of the individual classifiers nor the learning of the combination method, they lead to an underestimation of the performance. This suggests that clinically relevant adjustments of the definition of success and failure can result in increased accuracy of the combined engine. Comparative studies aiming at evaluating EuResist vs. state-of-the-art systems and expert opinion are under way.

The combined EuResist prediction system is freely available online at http://engine.euresist.org.
